# Treatment of Dental Caries, Etc.

**Published:** 1851-10

**Authors:** 


Treatment of Dental Caries Complicated with Disorders of the Pulp and Peridental Membrane.—We have published in the present number of the Register, the third and last article on this subject, from the American Journal, by Dr. R. Arthur.

The present article treats more particularly of that condition of decay, where "the necessary operations to arrest its progress, cannot be done without danger of injury to the pulp." The Doctor rather advocates in theory what has been long *practiced,* the leaving of decayed matter in the cavity, but more practiced than advocated. In volume VII No. 1, American Journal, page 74, there is an article on the Preparation of a Cavity in a Tooth, preparatory to Plugging, by Dr. Dwinelle, which was read before the American Society of Dental Surgeons, in which the Doctor takes the same ground, and remarks "that there are circumstances wherein it is not only proper but duty to leave discolored and even softened parts in the tooth. Dr. Dwinelle recommends the removal of all the decay except that portion the removal of which would expose the nerve, and we presume that Dr. Arthur would in all cases practice the same. Our experience has given us more confidence in the capping of the nerve and plugging than Dr. Arthur entertains; and we see so many teeth filled (unskilfully it is true,) without the carious portion having been well removed, that we dislike to leave dead matter in the cavity. There is often a stratum of diseased or inflamed bone covering the nerve, and interposed between it and the softened caries, which we would leave in preference to an utter exposure of the nerve. The earthy particles of the bone has not yet been disengaged by the chemical agent inducing decay. The most of this agent is cut off by the removal of the soft decayed matter. Such cavities it would be well to wash out, as Dr. Dwinelle remarks, "with a solution of soda." This inflamed bone will generally become darkened under the plug yet not softened if air and moisture is excluded, and we doubt not that very, often in such cases depositions of bony matter takes place and effectually shields the pulp. We cannot see how the cavity can be well filled without the walls are of sound and healthy bone.

In a former article Dr. Arthur speaks of the use of cobalt as an agent for the removal of sensibility of the tooth bone in filling teeth. Nine or ten years since, shortly after we settled in Cincinnati, there was much noise made about nerve killing, and one or two gentlemen who entered into the business most extensively, used a black powder, a small portion of which we had procured, and at first thought it was cobalt; but, on further inspection we found it to be arsenious acid mixed with charcoal. This led us, however, to test the cobalt and we found it to answer every purpose of the arsenic, and, as we thought, gave less pain and was less dangerous. We made oft and repeated experiments with it, as an agent for destroying the sensibility of the tooth bone, but have not as favorable an opinion of it as the Doctor. Several times it ultimately destroyed the nerve, and gave us the same trouble as the common arsenical preparations; yet, for destroying the nerve or sensibility of tooth bone, we regard it as better than any agent we have. In using it for the latter purpose we scarcely ever leave it in longer than three or four hours. We made known its use to the Cincinnati Association some nine years since, and it has been in use more or less west ever since.

